# Retentive Strength of CAD/CAM-Fabricated All-Ceramic Crowns Luted on Titanium Implant Abutments Using Different Ceramic Materials and Luting Agents: An In Vitro Study

**DOI:** 10.3390/ma15196968

**Published:** 2022-10-07

**Authors:** Monika Bjelopavlovic, Michael Weyhrauch, Herbert Scheller, Stefan Wentaschek, Karl Martin Lehmann

**Affiliations:** Department of Prosthetic Dentistry, University Medical Center Mainz, Augustusplatz 2, 55131 Mainz, Germany

**Keywords:** implant, ceramic, retentive strength, CAD/CAM, luting agent, cement

## Abstract

**Objectives:** This study aimed to determine the retentive strength of monolithic hybrid-all-ceramic crowns luted on titanium implant abutments. **Material and Methods:** In total, 450 crowns (75 each of Mark II, Empress CAD, e.max CAD, Suprinity, Enamic, Celtra Duo) were milled using a CAD/CAM system. The crowns were cemented onto sandblasted titanium implant abutments using five luting agents (Multilink Implant, Variolink II, RelyX Unicem, Fujicem, and Panavia 2.0). After thermocycling was performed (5000 cycles: 5–55 °C, 30-s dwell time), the crowns were removed using a universal testing machine. The location of luting-agent residue on the abutment and inner crown surfaces was evaluated. Analyses of variance (ANOVA) with the Bonferroni correction were performed to evaluate differences of retentive strength depending on the crown material and the kind of the luting agent. **Results:** The retentive strengths for the different ceramic materials were Vita Mark II: 652N-759N (SD:134N-146N), Empress CAD: 681N-822N (SD: 89N-146N), e.max CAD: 784N-1044N (SD: 109N-176N), Vita Enamic: 716N-1177N (SD: 132N-220N), Vita Suprinity: 867N-1488N (SD: 202N-278N), and Celtra Duo 772N-1335N (SD:151N-229N). After the removal trials, the visual documentation showed different adhesive residue location depending on the ceramic materials. Furthermore, the pull-off force was dependent on the choice of adhesives. No significant differences were found between different luting agents and the ceramic material Vita Mark II and Empress CAD. EmaxCAD showed significant differences with Unicem and FujiCem compared to Panavia, as did VitaSuprinity, VitaEnamic, and Celtra Duo (*p* < 0.001). **Conclusions:** The ceramic material used seems to influence the retentive strength and the use of certain luting agents results in a higher retentive strength for some ceramic materials.

## 1. Introduction

The request for ceramic fixed partial dentures is increasing because all-ceramic materials constitute an appropriate option, optimal esthetics, offering excellent biocompatibility and low thermal conductivity. In reference to this, digital workflows have the potential to reach an outcome with the production of ceramic restorations comparable to conventional procedures from the patient’s point of view [1,2,3]. Over the years, several ceramic systems have been developed and introduced, using various materials and techniques for CAD/CAM fabrication. In this context CAD/CAM systems were optimized regarding an adequate internal and marginal fitting [4,5], esthetics, stability, and functional aspects as relevant success factors. Regarding this a distinction can be made between screw-retained and cemented restorations and also with these systems the production of monolithic restorations are possible, which offer reduced chipping problems compared with restorations based on a core material layered with a veneering ceramic. For this several ceramic classes are used, i.e., feldspathic, glass and oxide ceramic materials and further hybrid-ceramics and different luting systems, namely, adhesive composite materials, non- and self-etching, self and light curing and a glass ionomer cement. These combinations of ceramic restorations and luting agents are not only used on teeth, but also utilized for implant-based restorations. The implant-based restorations could be screw-retained or consist of ceramic suprastructures cemented on abutments [6,7]. Each retention mode offers advantages, so screw retained implant based restorations avoid cement residues and thus biological implant pathologies [8] and guarantees a fixation when the vertical available space for the suprastructure is limited and thus does not offer a sufficiently large cementation surface. However, a fixation concept of a vertical screw retention cannot always be implemented, e.g., with a strong divergence between implant length axis and the axis of the prosthetic restoration regarding esthetic aspects, especially in visually exposed regions as the front teeth. In the case of cemented restorations an important aspect for long term success is a sufficient fixation of the ceramic suprastructures on the abutment platforms. Furthermore, a study showed that the stability of the crown-abutment connection in implants can be dependent on the “ferrule effect”, analogous to natural teeth, and consequently increases in the presence of ferrulization [9]. The fixation data published is as follows: analyzing the effect of contamination of the bond surface [10,11,12], the cement type and abutment height [13], abutment platform configuration [14], airborne-particle abrasion by using alumina [15] or silanisation and laser pretreatment [16] on retention force of the restoration. In addition, it is possibly to be expected that the adhesive interface behavior between the implant surface, the luting agent, and the bond surface of the ceramic restoration could be meaningful for the stabilization of the ceramic restoration. Currently, the adhesion strength between the upper part of the dental implant and the crown is not clear, and this study analyzes and explores previous adhesion.

In the present study the focus was primarily placed on the combination effects using different ceramic materials and luting agents on the retentive strength. With the help of the standardized pull-off test to reach a comparability with previous studies, the quality of the bonding was tested. This target size was accepted as a measure of long-term success using six ceramic materials in combination with five different luting systems regularly used clinically for the fixation of dental restorations.

## 2. Materials and Methods

For this study, two working hypotheses were formulated:

In terms of retentive strength,

1. all ceramic materials used would behave independently of the type of luting agent applied; and
2. all luting agents would result in equal values independent of the type of ceramic material.

In total, 450 implant abutments (No. 57120, Bego Implant Systems, Bremen, Germany) were screwed onto implant laboratory analogs (No. 56696, Bego Implant Systems, S-RI line, 4.1 mm diameter; 0.5 mm machined shoulder)) using a torque driver (No. 55799, Bego Implant Systems) with 25 Ncm. Seventy-five crowns of a mandibular right first premolar, made of ceramic materials (75 each of Vita Mark II; feldspar ceramic [FSC], Ivoclar Empress CAD; leucite reinforced glass ceramic [LrGC], Ivoclar e.max CAD; lithium disiclicate [LiDS], Vita Enamic; polymer reinforced fine-structure feldspar ceramic [PolyFSP], Vita Suprinity; pre-sintered zirconia reinforced lithium silicate ceramic [PSZirLS], Celtra Duo; fully crystallized zirconia reinforced lithium silicate [FcZirLS]); Table 1), were manufactured using a CAD/CAM system (CEREC inLab version 4.1; Sirona, Bensheim, Germany; CEREC parameters: block size 14 mm, cement space 30 μm, and spacer 0 μm).

The exterior surfaces of the implant abutments were sandblasted (corundum, 50 µm, ~10-mm distance, 1 bar, ~60-s blasting time per abutment), degreased, and bonded with silane (Monobond P; Ivoclar Vivadent Clinical, Schaan, Lichtenstein). The inner surfaces of the crowns were etched with hydrofluoric acid (5%; Ivoclar Vivadent Clinical) and silanized with Monobond P. The abutment and inner crown surfaces were also pretreated with Monobond P. This resulted in 15 different test series. All six ceramic materials and five luting agents used (Table 1) were applied in strict accordance with the manufacturers’ instructions (Table 2). All cement excesses were immediately removed with a sponge. The proprietary glycerine gel of each luting system was subsequently applied at the crown margin. If no such gel was available from the respective manufacturer, an alternative gel (Airblock) was applied. In the case of dual-curing systems crowns were polymerized using a polymerization light (Bluephase; Ivoclar Vivadent Clinical) for 240 s.

To simulate the oral environment, the implant-abutment-crown complexes were placed in a moist container for 30 min at 37 °C in an insulated chamber. After 30 min, specimens were placed in water for 1 week at 37 °C. Thereafter, thermocycling was performed for 5000 cycles (5–55 °C, 30-s dwell time; Willytec; SD Mechatronik GmbH, Feldkirchen-Westerham, Germany). All experiments were performed under a constant room temperature at 21 degrees.

To enable the crowns to be pulled off with the testing machine, it was necessary to create a well-fitting construction that would serve as an adapter between the all-ceramic crowns and the universal testing machine (Model 1425; Zwick Roell GmbH & Co. KG, Ulm, Germany). This resulted in an adapter that had an accurate and homogenous contact with the crowns.

All pull-off trials were conducted with the universal testing machine. The specimens were taken from the water bath prior to the pull-off test and mounted in the machine. The testing machine was adjusted so that the two tensile heads holding the abutment respective to the crown moved strictly vertically at a speed of 1 mm/min. After the crowns had been pulled off, the location of luting-agent residue was evaluated by visual observation.

Retentive strength was recorded, using SPSS 25.0 mean, standard deviations, minimum and maximum were calculated (Table 3). A Bonferroni adjustment was performed, and an analysis of variance was conducted to evaluate statistical differences with alpha adjustment. With level of significance α = 0.00011494.

## 3. Results

**Figure 1:**
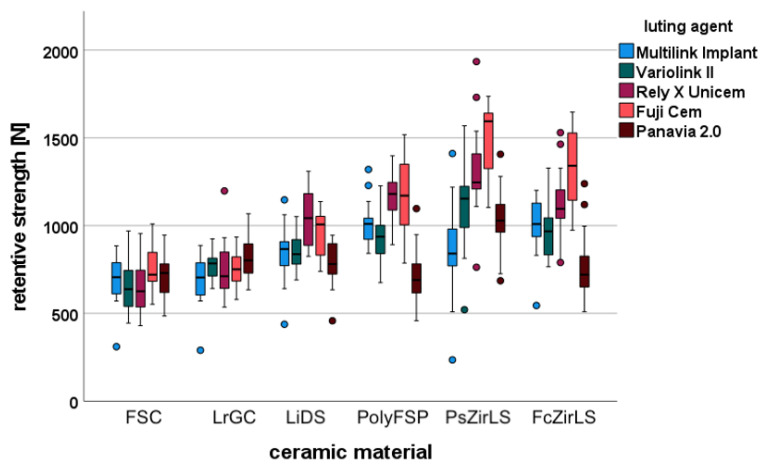
Retentive forces of various ceramic materials luted with different luting agents.

Most of the ceramics used, tended to show higher pull-off forces when using Unicem and FujiCem. **No significant differences between the luting agents for the ceramic materials FSC and LrGC were observed (Figure 1)**. For the material **LiDS a significant higher retentive strength for Unicem compared to Panavia** was observed (*p* < 0.0001). For ceramic **PolyFSP significant higher retentive strength** for the luting agents Multilink implant vs. Panavia 2.0, **Fujicem and Unicem** vs. Variolink and Unicem and Fujicem vs. Panavia were detected (*p* < 0.0001). The ceramic PsZirLs showed significantly higher retentive differences between the luting agents Unicem and Fujicem vs. Multilink implant, Fujicem vs. Variolink, and Fujicem vs Panavia (*p* < 0.0001). For ceramic **PcZirLS significant higher retentive strengths** were detected between the luting agents **Fujicem** vs. Multilink and Variolink, **Unicem** vs. Panavia, and Fujicem vs. Panavia (*p* < 0.0001).

With the focus on the luting agents the agent Multilink Implant showed a higher retentive strength in the combination with PolyFSP and FcZirLS vs. FSC, PolyFSP and FcZirLS vs. LrGC (*p* < 0.001); the agent Variolink II showed a higher retentive strength in combination with PolyFSP, PsZirlLs and PcZirLS, compared to FSC, for PsZirLS a higher retentive strength than for LrGC and for LiDS (*p* < 0.001); when the agent Unicem was used the combination with LiDS, PolyFSP, PsZirLS, and FcZirLS exhibit a higher retentive strength compared to FSC, further the combination of LiDS, PolyFSP, PsZirLS and FcZirLS a higher retentive strength compared to LrGC (*p* < 0.001), for the combination of PsZirLS a higher retentive strength than for LiDS (*p* < 0.001); for the luting agent Fujicem the combination with PolyFSP, PsZirLS and FcZirLS a higher retentive strength compared to FSC and LrGC, for PsZirLS and FcZirLS a higher retentive strength compared to LiDS and for PsZirLS compared to PolyFSP; for the luting agent Panavia 2.0 the combination with PSZirLS showed a higher retentive strength compared to FSC, PolyFSP and FcZirLS (*p* < 0.001) (Appendix A). The location of the luting agent’s residue is documented in Table 4.

## 4. Discussion

To satisfy patient demand for metal-free fixed partial dentures that provide long-term stability, it is necessary to keep several issues in mind. It is well known that in addition to marginal adaption, high fracture strengths, and sufficient aesthetics, one of the most important aspects for long-term survival of luted fixed partial dentures (i.e., crowns) is the fixation of the restoration achieved as a quality factor for long-term success [17]. Thus, in several studies the bond strength was used as a prognostic factor to evaluate clinical suitability, i.e., for veneer restorations on natural teeth [18]. However, the question arises as to how it behaves with ceramic restorations on titanium abutments. Therefore the experimental designs of those studies, just like in this study, are [19,20] to simulate the clinical situation, i.e., of crowns made of monolithic all-ceramic materials luted on implant abutments, and thus allowing a realistic assessment of the retentive strength as a factor for achievable long-term success when frequently used ceramic materials and luting agents are utilized. With regard to this, the retentive strength depends on several factors: among other things the texture of the retentive surfaces respective to their pretreatment [21,22,23,24], the luting agent or their curing mechanism [25,26], or the geometry of the retentive surfaces [27,28,29,30,31]. So special attention was paid to allow a sufficient dwell time of cemented crowns in 100% humidity before they were placed in water. For some luting agents, placement in water immediately after removing cement excesses can lead to rapid hydrolytic degradation of the bond (for example if the luting agent has not completely cured) and a reduction in the bonding potential of the luting agent [20,32]. This does not absolutely correspond with the clinical situation, in which the cement is in contact with saliva rather than water. However, in this study long-term water storage was not conducted because Ernst et al. [20] showed that it had no further significant effect on retentive strength, whereas thermocycling was carried out to simulate the thermal stress as a relevant factor for reducing bond strength [23,33,34]. In this study it was of value that representative and commonly used ceramic materials and luting agents were used. Different classes of ceramic materials, feldspathic, lithium disilicate, a hybrid ceramic, and ceramic materials with oxide ceramic components were utilized and regarding the luting agents also the different types which are frequently encountered in everyday clinical practice, such as adhesive composite materials, non- and self-etching, self and light curing, and a reinforced glass ionomer cement. Although manufacturers of ceramic materials and professional societies make specific recommendations for the combination of the ceramic materials and luting agents the question arises as to what extent other combinations influence the retentive strength. So, the first working hypothesis, that each ceramic material used would behave independently of the type of luting agent regarding the retentive strength, has only been accepted for the Mark II and Empress CAD ceramics. For the other ceramic materials using this hypothesis had to be rejected. The second hypothesis, that all luting agents would result in equal retentive values independent of the type of ceramic material had to be rejected completely.

To explain the results, it is necessary to analyze the fixation, on the one hand the interface between the ceramic surface and the luting agent and on the other hand between the luting agent and the adhesive surface of the implant abutment. In this regard the location of the residue of the luting agent allows the quality of the bonding to be judged and helps to understand the behavior of releasing the bond during the withdrawal process. Historically dental ceramics were first bonded on natural teeth and based on this there have already been numerous publications and findings on the bonding interface between ceramic material, luting agent, and the surface of natural teeth [34]. Compared to the experimental setup of these studies further studies were conducted where there was a titanium surface instead of human dentin.

The question is, whether there is a context between the location of the adhesive residue and the used luting agents and ceramic materials.

In this study regarding the feldspar ceramic and glass ceramic no significant differences between the luting agents were detected, the residues of the luting agents were primarily in the adhesive surfaces of the ceramic restoration and thus the prevailing adhesive failure was between the titanium surface and the luting agents and that showed a basically good adhesive connection between all luting materials to the feldspathic (Mark II) and glass ceramic (Empress) materials tested. For the lithium disilicate ceramic (LiDS) the luting agent Rely X Unicem showed a significant higher retentive strength compared to Panavia (*p* < 0.001) whereas the residues were also overwhelmingly in the inner crown surface. For the hybrid dental ceramic PolyFSP more significant differences between the luting agents were detected. So, for the luting agents Multilink implant, Rely X Unicem, and Fujicem a higher retentive strength was detected compared to Panavia 2.0, whereas it is noticeable that in addition to more significant differences between the luting agents for this hybrid ceramic materials more and more residue of the luting agents was located on the titanium abutment surface and less on the inner crown surface. For the pre-sintered zirconia-reinforced lithium silicate ceramic (PsZirLS) and the fully crystalized zirconia-reinforced lithium silicate ceramic (FcZirLS) higher retention values and higher standard deviations for nearly all combinations with the different luting agents were detected and the residue of the luting agents was also increasingly on the abutment surfaces.

With respect to the luting agents the self-polymerizing luting agent Multilink showed significantly higher retentive strength for the ceramic materials Enamic and Celtra Duo compared to the ceramics Mark II and Empress. For the luting agent Variolink as a dual-/light-polymerizing, resin-based dental luting material Enamic, Suprinity, and Celtra offered higher retentive strength compared to Mark II and Suprinity further to Empress. Regarding the dual-polymerizing, self-adhesive resin cement Unicem, the ceramic Empress, Enamic, Suprinity, and Celtra offered a significantly higher retentive strength compared to Mark II, while Enamic, Suprinity, and Celtra are also higher in retentive strength compared to the material Empress. Fujicem as a self-polymerizing, resin-reinforced glass-ionomer luting cement offer sa similar situation with a higher strength for Enamic, Suprinity, and Celtra in comparison to Mark II, and Empress and Suprinity, and Celtra higher than e.max and the material Suprinity higher than Enamic. In contrast the dual-polymerizing, self-adhesive resin cement Panavia showed only for Suprinity a higher retentive strength compared to Mark II.

The interpretation of these results is certainly difficult because despite all standardization of the experimental setup various influencing factors must be considered, essentially the different ceramic materials and luting agents. Because of the standardized setup the reason for this must be due to the factors ceramic material, luting agent, and the combination of them, the interface of the titanium surface, luting agent, and adhesive surface of the ceramic material. It is conspicuous that almost no significant difference between the luting agents in retentive strength for the feldspathic (FSC), the glass (LrGC), and the lithium disilicate (LiDS) material were noted. In summary the ceramic materials Enamic, Suprinity, and Celtra showed, in combination with the different luting agents tested, often relatively higher values for retentive strength compared to the feldspathic Mark II and the glass ceramic Empress. Increasingly more differences between the luting agents were detected for the hybrid (PolyFSP), the pre-sintered (PsZirLS) and fully crystalized (FcZirLS) zirconia-reinforced ceramic. Regarding these ceramic materials tested further studies stated also differences in fracture strength [35] with a higher fracture strength of hybrid (PolyFSP), the pre-sintered (PsZirLS), and fully crystalized (FcZirLS) zirconia-reinforced ceramic compared to the feldspathic (FSC) and the glass (LrGC) materials. It might indicate that the retentive strength could be related to the stability of the ceramic material. A hypothesis is that loosening of the crowns is caused by a failure of the adhesive connection initiated by microcracks that arise first in ceramic materials with lower stability. This could be an explanation for higher retention forces when ceramics with a higher stability are used. However, the question is further to explain the observed significant differences of retentive strength between crowns within each ceramic material made of the high stable materials (PolyFSP), (PsZirLS), and (FcZirLS). Those differences were probably caused by the different luting agents which for the crowns are made of materials mainly responsible for the formation of different adhesive connection patterns between the titanium surface, the layer of the luting agent, and the ceramic surface. However, it must be clarified as to what extent the results depend on individual components of the several luting agents used.

Limitations of this study are as follows:

(1) The choice of the conical implant with a machined implant shoulder could influence the results and was not compared to another implant system.
(2) The conditioning (HF) and bonding (Monobond S) are not part of the manufacturer recommended procedure for the cementation. Inclusion of these two steps may affect the comparison of results to other studies. Comparable groups should well be achieved within the study, however with the comparability to other studies being impaired.

In summary, the benefit of this study is that clinically the frequently used numerous ceramic materials and luting agent classes were tested in a standardized laboratory setup with corundum abrasion of the titanium abutments and completely identical testing procedures, with an adjacent consistent pull-off setup without the presence of influencing factors like saliva or blood, that could affect the quality of the fixation of the ceramic crowns. Further it is to be expected that the monolithic ceramic crowns made of the ceramic materials und luting agents used, will exceed physical masticatory forces, however certain combinations of ceramic materials and luting agents offer higher retentive strengths.

Thus, the first hypothesis that all ceramic materials behave independently of the type of luting agent applied was rejected for the materials LiDS, PolyFSP, PsZirLS, and FcZirLS and the second working hypothesis, that the luting agent used would result in equal retentive strength independent of the type of ceramic material was completely rejected.

## 5. Conclusions

Within the limitations of this study the following conclusions can be drawn:

(1) The ceramic material used influences the retentive strength of all ceramic crowns luted with various luting agents and
(2) the combination of certain ceramic materials with certain luting agents results in higher retentive strength.

Further investigation regarding adhesive residues should be the subject of subsequent studies on the effect and the adhesive mechanism of individual combinations of ceramic materials and luting agents.

## Figures and Tables

**Table 1:** Ceramic materials and luting agents tested in this study.

Ceramic Materials	Manufacturer	Manufacturer’s Site	Specification
Vita Mark II *[FSC]*	VITA Zahnfabrik H. Rauter GmbH & Co. KG	Bad Säckingen, Germany	Finely structured feldspar ceramic
Empress CAD *[LrGC]*	Ivoclar Vivadent	Schaan, Liechtenstein	Leucite-reinforced glass ceramic
e.max CAD *[LiDS]*	Ivoclar Vivadent	Schaan, Liechtenstein	Lithium disilicate glass ceramic
Vita Enamic *[PolyFSP]*	VITA Zahnfabrik H. Rauter GmbH & Co. KG	Bad Säckingen, Germany	Hybrid dental ceramic with polymer network 86% ceramic network 14% polymer network
Vita Suprinity *[PsZirLS]*	VITA Zahnfabrik H. Rauter GmbH & Co. KG	Bad Säckingen, Germany	Zirconia-reinforced lithium silicate pre-sintered 10% zirconia 90% lithium silicate
Celtra Duo *[FcZirLS]*	Dentsply Detrey GmbH	Konstanz, Germany	Zirconia-reinforced lithium silicate fully crystallized 10% zirconia 90% lithium silicate
Multilink Implant	Ivoclar Vivadent	Schaan, Liechtenstein	Self-polymerizing luting composite with light-curing option
Variolink II	Ivoclar Vivadent	Schaan, Liechtenstein	Dual-/light- polymerizing, resin-based dental luting material
Rely X Unicem	3M ESPE	Neuss, Germany	Dual-polymerizing, self-adhesive resin cement
GC Fujicem	GC Corporation	Tokyo, Japan	Self-polymerizing, resin-reinforced glass-ionomer luting cement
Panavia 2.0	Kuraray Europe GmbH	Hattersheim am Main, Germany	Dual-polymerizing, self-adhesive resin cement

**Table 2:** Conditioning of the ceramic material.

Material	Conditioning	Bonding
FSC	60 s, hydrofluoric acid	60 s, Monobond S
LrGC	60 s, hydrofluoric acid	60 s, Monobond S
LiDS	20 s, hydrofluoric acid	60 s, Monobond S
PsZirLS	60 s, hydrofluoric acid	60 s, Monobond S
PolyFSP	60 s, hydrofluoric acid	60 s, Monobond S
FcZirLS	30 s, hydrofluoric acid	60 s, Monobond S

**Table 3:** Retentive strength (mean, minimum, maximum, SD in N) divided by ceramic material and luting agent.

Ceramic Material	Luting Agent	*n*	Mean	Sd	Min	Max
FSC	MI	15	685	142	310	885
	VII	15	657	145	445	970
	RX	15	652	146	430	955
	GC	15	759	134	552	1010
	P	15	715	145	485	946
LrGC	MI	15	681	146	290	887
	VII	15	774	89	642	925
	RX	15	749	175	536	1198
	GC	15	748	98	579	935
	P	15	822	131	635	1068
LiDS	MI	15	842	176	438	1147
	VII	15	859	109	691	1052
	RX	15	1044	173	825	1310
	GC	15	970	135	740	1137
	P	15	784	133	458	945
PolyFSP	MI	15	1017	132	842	1320
	VII	15	919	150	675	1227
	RX	15	1169	142	892	1398
	GC	15	1177	220	787	1518
	P	15	716	169	458	1097
PsZirLS	MI	15	867	278	235	1411
	VII	15	1109	272	521	1569
	RX	15	1332	272	763	1935
	GC	15	1488	214	1104	1737
	P	15	1021	202	686	1407
FcZirLS	MI	15	1004	170	545	1201
	VII	15	969	151	766	1328
	RX	15	1130	204	790	1530
	GC	15	1335	229	974	1647
	P	15	772	209	510	1239

**Table 4:** Location of luting agents’ residue (MI: Multilink Implant, VII: Variolink II, RX: RelyX Unicem, GC: Fujicem, P: Panavia).

**Ceramic Material**		**FSC**					**LrGC**					**LiDS**				
**Luting Agent**		**MI**	**VII**	**RX**	**GC**	**P**	**MI**	**VII**	**RX**	**GC**	**P**	**MI**	**VII**	**RX**	**GC**	**P**
Location of luting agent residue: implant surface/inner crown surface	0%/100%	15	14	15	0	11	15	15	15	2	10	15	14	14	8	9
	25%/75%	0	1	0	7	4	0	1	0	8	5	0	1	1	4	6
	50%/50%	0	0	0	8	0	0	0	0	3	0	0	0	0	3	0
	75%/25%	0	0	0	0	0	0	0	0	2	0	0	0	0	0	0
	100%/0%	0	0	0	0	0	0	0	0	0	0	0	0	0	0	0
**Ceramic Material**		**PolyFSP**					**PsZirLS**					**FcZirLS**				
**Luting Agent**		**MI**	**VII**	**RX**	**GC**	**P**	**MI**	**VII**	**RX**	**GC**	**P**	**MI**	**VII**	**RX**	**GC**	**P**
Location of luting agent residue: implant surface/inner crown surface	0%/100%	0	1	2	0	1	0	0	0	0	0	0	0	0	0	0
	25%/75%	13	6	9	1	9	3	14	5	1	0	2	12	8	5	2
	50%/50%	0	0	3	2	2	5	0	2	1	4	2	2	2	3	2
	75%/25%	0	2	0	2	1	3	0	1	8	2	1	0	1	2	1
	100%/0%	2	6	1	10	2	4	1	7	5	9	10	1	4	5	10

**Figure A1:**
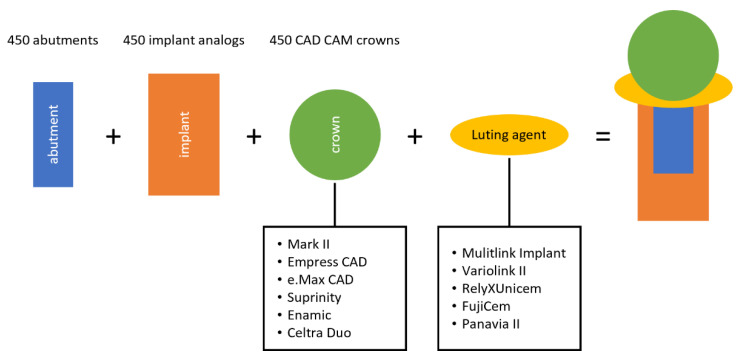
Schematic representation of the methodology.

## Data Availability

Data can be seen in Table 1, Table 2, Table 3 and Table 4.

## Appendix A

**VITA MARK II** [FSC]

**Table A1:** Comparison of the different adhesives during the pull-off test: Vita Keramik II.

	Multilink Implant	Variolink II	Rely X Unicem	Fuji Cem	Panavia 2.0
Multilink Implant		1.000	1.000	1.000	1.000
Variolink II	1.000		1.000	0.437	1.000
Rely X Unicem	1.000	1.000		0.437	1.000
Fuji Cem	1.000	0.437	0.437		1.000
Panavia 2.0	1.000	1.000	1.000	1.000	

**Ivoclar Empress CAD**[LrGC]

**Table A2:** Comparison of the different adhesives during the pull-off test: Ivoclar Empress CAD.

	Multilink Implant	Variolink II	Rely X Unicem	Fuji Cem	Panavia 2.0
Multilink Implant		0.590	1.000	1.000	0.047
Variolink II	0.590		1.000	1.000	1.000
Rely X Unicem	1.000	1.000		1.000	1.000
Fuji Cem	1.000	1.000	1.000		1.000
Panavia 2.0	0.047	1.000	1.000	1.000	

**Ivoclar e.max CAD** [LiDS]

**Table A3:** Comparison of the different adhesives during the pull-off test: Ivoclar e.max CAD.

	Multilink Implant	Variolink II	Rely X Unicem	Fuji Cem	Panavia 2.0
Multilink Implant		1.000	0.003	0.196	0.047
Variolink II	1.000		0.010	0.424	1.000
Rely X Unicem	0.003	0.010		1.000	0.000 *
Fuji Cem	0.196	0.424	1.000		0.009
Panavia 2.0	1.000	1.000	0.000 *	0.009	

* significant.

**Vita Enamic** [PolyFSP]

**Table A4:** Comparison of the different adhesives during the pull-off test: Vita Enamic.

	Multilink Implant	Variolink II	Rely X Unicem	Fuji Cem	Panavia 2.0
Multilink Implant		1.000	0.141	0.098	0.000 *
Variolink II	1.000		0.001	0.001	0.013
Rely X Unicem	0.141	0.001		1.000	0.000 *
Fuji Cem	0.098	0.001	1.000		0.000 *
Panavia 2.0	0.000 *	0.013	0.000 *	0.000 *	

* significant.

**Vita Suprinity** [PsZirLS]

**Table A5:** Comparison of the different adhesives during the pull-off test: Vita Suprinity.

	Multilink Implant	Variolink II	Rely X Unicem	Fuji Cem	Panavia 2.0
Multilink Implant		0.097	0.000 *	0.000 *	0.956
Variolink II	0.097		0.174	0.001	1.000
Rely X Unicem	0.000 *	0.174		0.906	0.011
Fuji Cem	0.000 *	0.001	0.906		0.000 *
Panavia 2.0	0.956	1.000	0.011	0.000 *	

* significant.

**Celtra Duo** [FcZirLS]

**Table A6:** Comparison of the different adhesives during the pull-off test: Celtra Duo.

	Multilink Implant	Variolink II	Rely X Unicem	Fuji Cem	Panavia 2.0
Multilink Implant		1.000	0.801	0.000 *	0.016
Variolink II	1.000		0.263	0.000 *	0.069
Rely X Unicem	0.801	0.263		0.053	0.000 *
Fuji Cem	0.000 *	0.000 *	0.053		0.000 *
Panavia 2.0	0.016	0.069	0.000 *	0.000 *	

* significant.

**Multilink Implant**

**Table A7:** Comparison of the different ceramics during the pull-off test: Multilink Implant.

	Vita Mark II	Ivoclar Empress CAD	Ivoclar e.max CAD	Vita Enamic	Vita Suprinity	Celtra Duo
Vita Mark II		1.000	0.291	0.000 *	0.094	0.000 *
Ivoclar Empress CAD	1.000		0.254	0.000 *	0.081	0.000 *
Ivoclar e.max CAD	0.291	0.254		0.128	1.000	0.227
Vita Enamic	0.000 *	0.000 *	0.128		0.384	1.000
Vita Suprinity	0.094	0.081	1.000	0.384		0.642
Celtra Duo	0.000 *	0.000 *	0.227	1.000	0.642	

* significant.

**Variolink II**

**Table A8:** Comparison of the different ceramics during the pull-off test: Variolink II.

	Vita Mark II	Ivoclar Empress CAD	Ivoclar e.max CAD	Vita Enamic	Vita Suprinity	Celtra Duo
Vita Mark II		0.832	0.013	0.000 *	0.000 *	0.000 *
Ivoclar Empress CAD	0.832		1.000	0.302	0.000 *	0.024
Ivoclar e.max CAD	0.013	1.000		1.000	0.001	1.000
Vita Enamic	0.000 *	0.302	1.000		0.032	1.000
Vita Suprinity	0.000 *	0.000 *	0.001	0.032		0.382
Celtra Duo	0.000 *	0.024	1.000	1.000	0.382	

* significant.

**Rely X Unicem**

**Table A9:** Comparison of the different ceramics during the pull-off test: RelyX Unicem.

	Vita Mark II	Ivoclar Empress CAD	Ivoclar e.max CAD	Vita Enamic	Vita Suprinity	Celtra Duo
Vita Mark II		1.000	0.000 *	0.000 *	0.000 *	0.000 *
Ivoclar Empress CAD	1.000		0.001	0.000 *	0.000 *	0.000 *
Ivoclar e.max CAD	0.000 *	0.001		1.000	0.001	1.000
Vita Enamic	0.000 *	0.000 *	1.000		0.352	1.000
Vita Suprinity	0.000 *	0.000 *	0.001	0.352		0.069
Celtra Duo	0.000 *	0.000 *	1.000	1.000	0.069	

* significant.

**Fuji Cem**

**Table A10:** Comparison of the different ceramics during the pull-off test: Fuji Cem.

	Vita Mark II	Ivoclar Empress CAD	Ivoclar e.max CAD	Vita Enamic	Vita Suprinity	Celtra Duo
Vita Mark II		1.000	0.017	0.000 *	0.000 *	0.000 *
Ivoclar Empress CAD	1.000		0.010	0.000 *	0.000 *	0.000 *
Ivoclar e.max CAD	0.017	0.010		0.022	0.000 *	0.000 *
Vita Enamic	0.000 *	0.000 *	0.022		0.000 *	0.247
Vita Suprinity	0.000 *	0.000 *	0.000 *	0.000 *		0.291
Celtra Duo	0.000 *	0.000 *	0.000 *	0.247	0.291	

* significant.

**Panavia 2.0**

**Table A11:** Comparison of the different ceramics during the pull-off test: Panavia 2.0.

	Vita Mark II	Ivoclar Empress CAD	Ivoclar e.max CAD	Vita Enamic	Vita Suprinity	Celtra Duo
Vita Mark II		1.000	1.000	1.000	0.000 *	1.000
Ivoclar Empress CAD	1.000		1.000	1.000	0.019	1.000
Ivoclar e.max CAD	1.000	1.000		1.000	0.002	1.000
Vita Enamic	1.000	1.000	1.000		0.000 *	1.000
Vita Suprinity	0.000 *	0.019	0.002	0.000 *		0.001
Celtra Duo	1.000	1.000	1.000	1.000	0.001	

* significant.
